# Longitudinal associations between food fussiness and parental feeding behaviors in Chinese children: between- and within-person effects

**DOI:** 10.1186/s12966-025-01830-8

**Published:** 2025-11-04

**Authors:** Fangge Qu, Yujia Chen, Xinyi Song, Xiaoxue Wei, Zhihui Zhao, Chenjun Wu, Ruxing Wu, Jian Wang, Xianqing Tang, Jinjin Chen, Daqiao Zhu

**Affiliations:** 1 School of Nursing, Shanghai Jiao Tong University, Shanghai, China; 2 School of Nursing and Health Management, Shanghai University of Medicine & Health Sciences, Shanghai, China; 3 School of International Medical Technology Nursing Department, Sanda University, Shanghai, China; 4 Department of Hematology and Oncology, Shanghai Children’s Medical Center, Shanghai Jiao Tong University School of Medicine, Shanghai, China; 5 Florence Nightingale Faculty of Nursing, Midwifery and Palliative Care, King’s College London, London, UK; 6 Department of Children’s Disease Prevention, Shanghai Pudong New Area Jinyang Community Health Service Center, Shanghai, China; 7 Department of Child Health Care, Shanghai Jiao Tong University Children’s Hospital, Shanghai, China

**Keywords:** Early childhood, Food fussiness, Feeding behaviors, Temperament, Sex, Cross-lagged analysis, Moderating effects

## Abstract

**Background:**

The directionality of longitudinal associations between children’s food fussiness and parental feeding behaviors remains contested. This study aimed to assess the dynamic relationship between children’s food fussiness and feeding behaviors.

**Methods:**

To disentangle these effects, this study employed cross-lagged panel models (CLPMs) and random-intercept cross-lagged panel models (RI-CLPMs) using longitudinal data from 588 Chinese children (Mean age = 3.7 years, SD = 0.3, 51.7% boys) across three waves over two years. CLPMs capture between-person associations, while RI-CLPMs isolate within-person dynamics over time. Within-person effects represent how temporary deviations predict subsequent changes beyond stable traits, whereas between-person effects reflect enduring cross-family differences.

**Results:**

Analyses revealed distinct patterns depending on the feeding behavior and model type: for restrictions, the CLPM showed parent-driven effects (restrictions at 3.7 years→ fussiness at 4.8 years, *β* = −0.104, *p* = 0.003), whereas the RI-CLPM identified child-driven effects (fussiness at 4.8 years → restrictions at 5.7 years, *β* = 0.179, *p* = 0.033). Both models consistently revealed child-driven effects for pressure to eat (CLPM: *β* = 0.151, *p* = 0.002; RI-CLPM: *β* = 0.218, *p* = 0.013). Food as a reward showed bidirectionality in CLPM (reward at 4.8 years → fussiness at 5.7 years: *β* = 0.112, *p* < 0.001; fussiness at 4.8 years→ reward at 5.7 years: *β* = 0.144, *p* = 0.005) but no significant cross-lagged paths in the RI-CLPM. Notably, the multi-group analysis revealed no moderating effect of child sex.

**Conclusions:**

After accounting for stable between-person differences, RI-CLPM findings reveal that child food fussiness prospectively drives increases in parental use of restriction and pressure to eat at the within-person level. This suggests that these specific feeding behaviors may function more as reactive responses to children’s eating behaviors than as caregiver-initiated strategies.

**Supplementary Information:**

The online version contains supplementary material available at 10.1186/s12966-025-01830-8.

## Introduction

 Food fussiness, characterized by children’s selective preferences and aversions toward specific foods, typically manifests as a reluctance to consume familiar foods or try novel foods [1]. This behavior is prevalent among preschool-aged children across both developed and developing nations. Childhood food fussiness is associated with poorer diet quality during childhood [2] and predicts food neophobia, emotional undereating, and unhealthy dietary patterns in adulthood [3, 4]. The development of children’s eating behaviors is shaped by multilevel environmental influences, with parental feeding behaviors constituting a core proximal factor that exerts immediate and sustained impacts on early eating patterns [5]. Empirical research has identified non-responsive feeding behaviors, including restrictions, pressure to eat and food as a reward, as risk factors for child food fussiness [6, 7]. From a biopsychosocial perspective, Russell & Russell (2019) proposed parent-child feeding interactions as dynamic transactional processes within family microsystems, where caregivers’ behaviors both shape and respond to children’s eating patterns [8]. Compared with cross-sectional designs, longitudinal designs facilitate temporal analysis of these dynamic interactions, providing stronger causal inference through repeated measurements. Recent studies have implemented multi-wave investigations examining the directionality and mechanisms underlying caregiver-child feeding interactions [9, 10].

Longitudinal studies examining the associations between child food fussiness and parental feeding behaviors have yielded mixed evidence, with some studies reporting bidirectional effects [11], whereas others highlight parent-driven effects or child-driven effects [12]. This inconsistency may be partially explained by child‒level moderators, particularly biological sex, which is a critical factor shaping longitudinal parent-child feeding dynamics. Research has revealed two sex-differentiated patterns: boys exhibit significantly higher levels of food fussiness than girls do [13], whereas parents tend to adopt sex-differentiated feeding strategies, such as employing more restrictive or controlling feeding behaviors with boys [14, 15]. These findings suggest that biological sex may serve as a moderating variable in caregiver‒child feeding dynamics, however, existing studies predominantly treat sex as a covariate rather than testing its moderating effect in reciprocal parent‒child feeding transactions.

In addition to moderating factors, these discrepancies may arise from methodological variations in statistical modeling. For example, multiwave analyses using different statistical approaches on the same dataset can lead to model-dependent conclusions. Multivariate linear regressions treating feeding behaviors and food fussiness as separate outcomes suggest reciprocal relationships between instrumental feeding (e.g., food as a reward) and food fussiness, while cross-lagged panel models (CLPMs) applied to the same data identify only unidirectional effects [16]. The CLPM has become the predominant method for analyzing reciprocal relationships, surpassing conventional regression techniques by simultaneously modeling variables’ autoregressive properties and bidirectional pathways [17]. However, a critical limitation of the CLPM is its conflation of between-person differences (i.e., stable individual traits) with within-person state fluctuations, which can lead to an ecological fallacy where population-level associations are misinterpreted as intraindividual causal processes [18].

To address this limitation, the random intercept cross-lagged panel model (RI-CLPM) incorporates random intercepts to capture stable between-person characteristics, thus isolating within-person dynamics by partialling out trait-level associations between constructs [18]. Empirical comparisons have shown that the traditional CLPM may overestimate cross-lagged effect estimates relative to the RI-CLPM [19]. Current methodological guidelines recommend that CLPM remains suitable for investigating population-level developmental trends, while the RI-CLPM provides a more robust analytical framework for examining within-person processes or deriving causal inferences from longitudinal data [20].

To address these gaps, the present study employed both the CLPM and the RI-CLPM to compare the between-person and within-person associations between child food fussiness and parental feeding behaviors. Additionally, multigroup CLPM and RI-CLPM were conducted to examine whether child sex moderated these longitudinal relationships.

## Method

### Participants

This study conducted a two-year longitudinal investigation across all eight kindergartens affiliated with a community health service center conveniently selected from the Pudong New District of Shanghai, China. The inclusion criteria were as follows: (1) children enrolled in target kindergartens’ entry-level classes serving children aged 3–4 years at baseline; (2) primary caregivers (father or mother) with a comprehensive understanding of children’s eating behaviors, as assessed through a targeted questionnaire item; and (3) the absence of communication barriers. The exclusion criteria were as follows: (1) children with genetic metabolic diseases, digestive system disorders, congenital diseases, intellectual disabilities, or attention-deficit/hyperactivity disorders; and (2) children who experienced acute/chronic diseases affecting appetite within the past month.

The research team trained pediatric healthcare providers from a community hospital and kindergarten health instructors to serve as investigators. Prior to formal data collection, investigators assigned unique identification codes to each participant to ensure the anonymity of longitudinal data. Parent-completed baseline paper questionnaires and informed consent forms were administered in December 2020, with follow-up data collected using questionnaires completed by the same parent during annual health examinations in December 2021 and December 2022.

## Measures

### Child food fussiness

Child food fussiness was assessed via the Food Fussiness subscale from the Chinese Preschooler’s Eating Behavior Questionnaire [21]. This subscale consists of five items, such as “My child will only eat foods they choose themselves”. Responses were recorded on a 5-point Likert scale (1 = “Never” to 5 = “Always”). The mean score across items represented the final score, with higher scores indicating greater food fussiness. The Cronbach’s α coefficients across the three time points were 0.74, 0.78, and 0.72, respectively.

### Parental feeding behaviors

Parental feeding behaviors were assessed using three subscales from the Chinese version of the Child Feeding Questionnaire [22], including restrictions (4 items, e.g., “I have to be sure that my child does not eat too many high fat foods”), pressure to eat (3 items, e.g., “If my child says ‘I’m not hungry’, I try to get her to eat anyway.”), and food as a reward (2 items, e.g., “I offer my child her favorite foods in exchange for good behavior.”). Each item is rated on a 5-point Likert scale (1 = “Never” to 5 = “Always”). Subscale scores were calculated as the mean of the item scores, with higher values indicating more frequent use of the corresponding feeding behaviors. The Cronbach’s α coefficients across the three waves were as follows: restrictions (0.75, 0.81, 0.77), pressure to eat (0.73, 0.77, 0.79) and food as a reward (0.63, 0.57, 0.60).

### Covariates

The study collected demographic data, including the children’s sex, age, height, weight, parental education levels and annual household income. Trained investigators used calibrated stadiometers and digital scales to measure the height and weight of children. Other data were obtained through parental questionnaires. The children’s BMI-for-age Z-scores (BAZ) were computed using WHO Anthro 3.2.2 and WHO Anthro Plus 1.0.4 software, and categorized according to WHO standards [23]: underweight (Z-score < − 2), normal weight (− 2 ≤ Z-score < 1) and overweight/Obesity (Z-score ≥ 1).

### Statistical analysis

The Data were analyzed using RStudio (version 4.3.0). Normality tests were performed to assess data distribution. Spearman’s rank correlation analysis was used to examine the correlations between child food fussiness and parental feeding behaviors.

The CLPMs and RI-CLPMs were constructed using the *lavaan* package. CLPM examines whether individuals with higher levels of a construct than their peers predict subsequent between-person differences in another construct. RI-CLPM evaluates whether deviations from one’s typical level predict subsequent within-person changes, after accounting for stable traits through random intercepts [20]. Model parameters were estimated using maximum likelihood estimation (MLE), and model comparisons were conducted using chi-square difference tests. Missing data were handled with full information maximum likelihood (FIML), ensuring that participants with baseline data were retained in model analyses regardless of follow-up participation.

Model fit was evaluated using the following indices: the chi-square to degrees of freedom (*χ²/df*), the comparative fit index (CFI), the Tucker‒Lewis index (TLI), the root mean square error of approximation (RMSEA) and the standardized root mean square residual (SRMR). The acceptable fit thresholds were: *χ²/df* ≤ 5, CFI ≥ 0.90, TLI ≥ 0.90, RMSEA ≤ 0.08, and SRMR ≤ 0.10 [24].

Multigroup CLPM and RI-CLPM analyses were conducted to examine the moderation effects of child sex. Likelihood ratio tests (*Δχ²*) compared constrained models (with equal cross-group path coefficients) with unconstrained models (with freely estimated path coefficients). A statistically significant *Δχ²* (*p* < 0.05) indicated significant moderation by sex.

## Results

The study initially recruited 644 participants at baseline. After screening for invalid questionnaires (e.g., missing key variables or patterned responses defined as systematic alternating responses [e.g., 1-2-1-2 pattern]) and applying the exclusion criteria, the baseline sample included 611 mothers or fathers and their children (94.8% of the recruited sample). Longitudinal follow-up assessments retained 471 participants at the first follow-up (T2, 12-month, 77.1% retention) and 482 participants at the second follow-up (T3, 24-month, 78.9% retention).

**Fig. 1:**
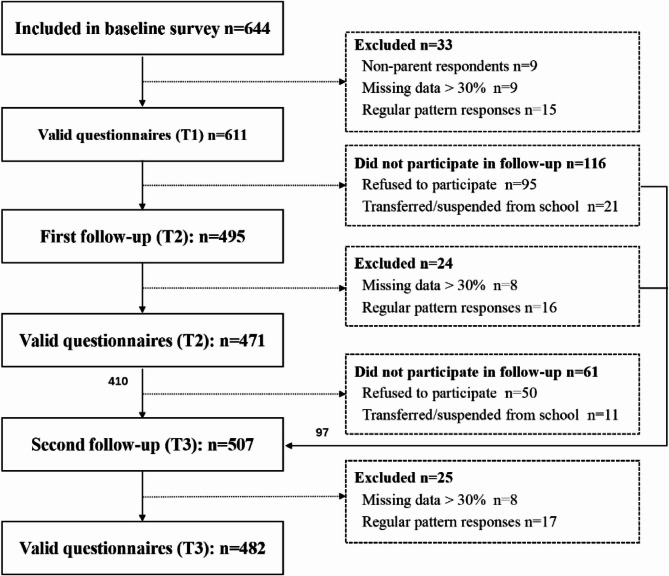
Flow chart of participation in longitudinal study

The detailed tracking status is presented in Fig. 1.

### Participant characteristics

At baseline, the mean age of the children was 3.7 years(SD = 0.3), with boys comprising 51.7% of the sample. The prevalence of overweight or obesity among the children was 15.4%, and 77.1% of the families reported mothers as primary caregivers (see Table 1 for full demographic characteristics). Intraclass correlation coefficients (ICCs) across the three waves indicated that 52% of the variance in child food fussiness originated from between-person differences, while 48% was attributed to within-person fluctuations. Parental feeding behaviors exhibited ICCs ranging from 32% to 43%, suggesting that 57%–68% of the variance in these behaviors was due to within-person variability (Table 2). These findings highlighted significant temporal variability in both children’s food fussiness and parental feeding behaviors, supporting the use of the RI-CLPM to distinguish between and within-person effects for a more precise mechanistic exploration.

Children’s food fussiness and parental feeding behaviors showed significant autocorrelations across time points (*r* = 0.354–0.643, *p* < 0.001). Significant cross-sectional (*r* = − 0.178–0.643, *p* < 0.001) and lagged correlations (*r* = 0.099–0.250, *p* < 0.001) were observed among the variables at adjacent waves (Supplementary Table S1).

**Table 1:** Demographic characteristics of the study participants

Variables	T1 (*n* = 611)		T2 (*n* = 471)			T3 (*n* = 482)		
	*n* (%)/mean (SD)	Missing(%)	*n (%)/mean (SD)*	Missing(%)		*n (%)/mean (SD)*	Missing(%)	
Children characteristics								
Age (years)	3.7 (0.3)	12 (2.0)	4.8 (0.3)	1 (0.2)		5.7 (0.3)	1 (0.2)	
Sex (%Boys)	316 (51.7)	NA	252 (53.5)	NA		246 (51.0)	NA	
BAZ	0.15 (0.92)	12 (2.0)	0.26 (0.88)	4 (0.8)		0.20 (1.10)	3 (0.6)	
Overweight or obese	92 (15.40)	12 (2.0)	74 (15.70)	4 (0.8)		96 (19.90)	3 (0.6)	
Parental characteristics								
Fathers’ age	36.55 (4.78)	23 (3.8)	37.69 (4.56)	19 (4.0)		38.28 (4.60)	16 (3.3)	
Mothers’ age	34.54 (3.89)	22 (3.6)	35.69 (3.98)	19 (4.0)		36.41 (3.82)	18 (3.7)	
Fathers’ educational level: college or higher	425 (88.2)	20 (3.3)	418 (88.7)	12 (2.5)		435 (90.2)	7 (1.5)	
Mothers’ educational level: college or higher	425 (88.2)	18 (3.0)	416 (88.3)	12 (2.5)		435 (90.2)	7 (1.5)	
Family factors								
The primary caregiver is the mother	471 (77.1)	NA	349 (74.1)	16 (3.4)		344 (71.4)	9 (1.9)	
Annual family income <300,000 CNY	240 (41.7)	35 (5.7)	157 (33.3)	35 (7.4)		164 (34.0)	24 (5.0)	
> 500,000 CNY	134 (23.3)		118 (25.1)			128 (26.6)		

*SD* standard deviation, *NA* not applicable, *BAZ* body mass index-for-age z-score, *T1* Time 1, *T2* Time 2, *T3* Time 3

**Table 2:** Descriptive statistics of study variables [Mean (SD)]

Variable scores	T1 (*n* = 611)	T2 (*n* = 471)	T3 (*n* = 482)	ICC(95% CI)	F
Food fussiness	3.07 (0.68)	2.87 (0.72)	2.79 (0.68)	0.52 [0.48,0.57]	4.3^***^
Restrictions	3.48 (0.76)	3.67 (0.74)	3.59 (0.74)	0.37 [0.32,0.42]	2.8^***^
Pressure to eat	3.25 (0.78)	3.08 (0.80)	2.86 (0.83)	0.43 [0.38,0.48]	3.3^***^
Food as a reward	3.32 (0.79)	3.39 (0.79)	3.16 (0.81)	0.32 [0.27,0.37]	2.4^***^

*SD* standard deviation, *ICC* 95% *CI* 95% confidence interval, ^***^P < 0.001, T1: Time 1, T2: Time 2, T3: Time 3

### Bidirectional models of child food fussiness and parental feeding behaviors

**Fig. 2:**
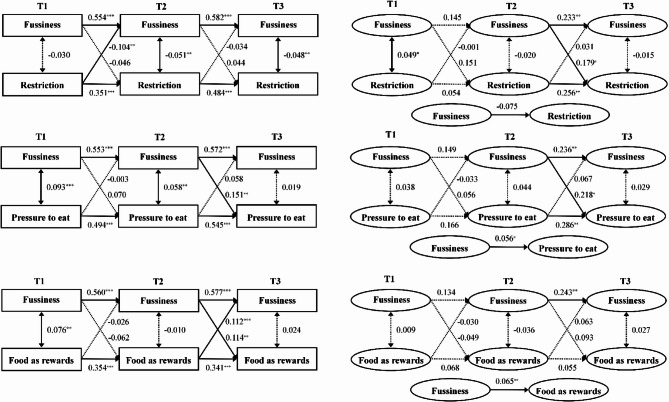
Cross-lagged panel model and random intercept cross-lagged panel model of children’s food fussiness with parental feeding behaviors: restriction (a), pressure to eat (b), and food as a reward (c) Note. ^*^p < 0.05, ^**^p < 0.01, ^***^p < 0.01. Bolded paths are significant at *p* < 0.05, and non-significant paths are shown with a dotted line. T1: baseline, T2: one year later, T3: two years later. CLPM: cross-lagged panel model, RI-CLPM: random intercept cross-lagged panel model. Note ^*^p < 0.05, ^**^p < 0.01, ^***^p < 0.01. Bolded paths are significant at *p* < 0.05, and non-significant paths are shown with a dotted line. T1: baseline, T2: one year later, T3: two years later. CLPM: cross-lagged panel model, RI-CLPM: random intercept cross-lagged panel model.

Compared with the CLPM, the RI-CLPM demonstrated superior model fit (Supplementary Table S2), with diverging results between the models. While all variables in the CLPM exhibited significant autoregressive effects across waves, within-person autoregressive effects in the RI-CLPM were observed only for food fussiness, restrictions and pressure to eat from T2 to T3 (Fig. 2).

For restriction, the models yielded opposing directional effects. The CLPM supported a parent-driven effect: children who received higher restriction than peers at T1 showed greater subsequent reduction in food fussiness at T2(*β* = −0.104, 95% CI [− 0.173, − 0.035], *p* = 0.003). Conversely, the RI-CLPM revealed a child-driven effect: when a child exhibited higher fussiness than their own average at T2, parents subsequently increased restriction beyond their typical usage at T3 (*β* = 0.179, 95% CI [0.014, 0.165], *p* = 0.033).

In analyses of parental pressure to eat, both models consistently identified child-driven effects. Higher food fussiness at T2 significantly predicted increased pressure to eat at T3 in both the CLPM (*β* = 0.151, 95% CI [0.057, 0.245], *p* = 0.002) and the RI-CLPM (*β* = 0.218, 95% CI [0.047, 0.389], *p* = 0.013).

For food as a reward, the CLPM demonstrated bidirectional cross-lagged effects: Higher food as a reward than peers at T2 predicted increased food fussiness at T3 (*β* = 0.112, 95% CI [0.049, 0.175], *p* < 0.001), while higher food fussiness than peers at T2 also predicted increased food as a reward at T3 (*β* = 0.144, 95% CI [0.044, 0.244], *p* = 0.005). However, no significant cross-lagged paths emerged in the RI-CLPM (Supplementary Tables S3–S5).

At the between-person level of the RI-CLPM, random intercepts for parental pressure to eat (*β* = 0.056, 95% CI [0.009, 0.103], *p* = 0.024) and food as a reward (*β* = 0.065, 95% CI [0.024, 0.106], *p* = 0.002) showed significant positive associations with the random intercept of child food fussiness. Adjusted models that included covariates (child sex, weight status, parental education and household income) produced consistent results, confirming the robustness of these findings (Supplementary Tables S3–S5).

### Moderating effect of child sex

Neither the CLPM nor the RI-CLPM demonstrated significant moderating effects of child sex. For restriction, nested model comparisons between boys and girls revealed no statistically significant differences (CLPM: *Δχ²/Δdf* = 0.606, *p* = 0.774; RI-CLPM: *Δχ²/Δdf* = 0.653, *p* = 0.733). Similar non-significant results were observed for pressure to eat (CLPM: *Δχ²/Δdf* = 1.001, *p* = 0.426; RI-CLPM: *Δχ²/Δdf* = 0.997, *p* = 0.436) and food as a reward (CLPM: *Δχ²/Δdf* = 1.003, *p* = 0.431; RI-CLPM: *Δχ²/Δdf* = 1.156, *p* = 0.322). These results indicated that child sex did not significantly moderate the longitudinal associations between feeding behaviors and food fussiness across three measurement waves (Supplementary Tables S6–S8).

### Sensitivity analysis

To evaluate the robustness of our findings to missing data assumptions, we conducted supplementary analyses using the subsample of participants with complete data across all three waves (*n* = 389). The RI-CLPM was re-estimated following identical specifications to our primary FIML approach. Results demonstrated remarkable consistency across key parameters (Supplementary Tables S3–S5).

## Discussion

This two-year longitudinal study with three measurement waves utilized both the CLPM and the RI-CLPM to investigate the bidirectional relationships between child food fussiness and parental feeding behaviors, while also examining the moderating effect of child sex. The CLPM identified more significant cross-lagged paths than the RI-CLPM did, whereas the RI-CLPM exhibited superior model fit. Significant associations between between-person random intercepts in the RI-CLPM suggested that neglecting between-person heterogeneity might lead to inflated cross-lagged effect estimations. The RI-CLPM results primarily supported child-driven effects, specifically demonstrating that child food fussiness predicted subsequent increases in parental restriction and pressure to eat. No significant moderation effects by sex were observed.

Child food fussiness and parental feeding behaviors showed significant stability over time during the preschool years, as indicated by moderate-to-strong autoregressive effects. Simultaneously, meaningful within-person fluctuations were observed, supported by intraclass correlations and temporal autocorrelations. This combination of stability and variability aligns with prior research [25, 26]. For instance, Powell (2018) reported correlation coefficients ranging from 0.43 to 0.81 between observed and mother-reported eating behaviors in a longitudinal study of 2- to 4-year-olds [26]. Both Powell (2018) and Farrow (2012) identified significant temporal variability in parental pressure to eat, whereas other feeding behaviors remained relatively stable [25, 26].

The RI-CLPM analysis revealed significant between-person differences, as indicated by correlated random intercepts in this study. This finding demonstrates that child food fussiness and parental feeding behaviors exhibit distinct family-specific characteristics. Specifically, different families or individuals maintain relatively stable behavioral patterns, which are likely attributable to heterogeneous family backgrounds, parenting styles, or other contextual factors [27]. Methodologically, this finding validates the dual-level analytical capability of the RI-CLPM: it simultaneously captures within-person dynamic associations across time (first-level relationships) while disentangling persistent between-person structural differences (second-level relationships). This hierarchical approach provides an essential methodological framework for elucidating complex variable interactions in developmental studies.

At the dimensional level, the RI-CLPM revealed significant positive between-person correlations between parental pressure to eat and food as a reward with child food fussiness. This finding aligned with prior evidence [11, 12] that parental self-reports of frequent pressure to eat and food as a reward were associated with increased child food fussiness. Notably, this study revealed significant negative correlations between between-person differences in parental restriction and child food fussiness. This showed inconsistencies with existing literature: while some studies reported no significant associations [12], others reported positive correlations [28]. A meta-analysis by Werner & Mallan (2024) suggests that restriction may not adversely affect child eating behaviors, potentially demonstrating null or even beneficial effects [29].

This discrepancy may arise from the distinction between restrictions’ subtypes. Researchers have proposed differentiating overt restrictions (e.g., explicit prohibitions or portion control) from covert restrictions (e.g., subtle environmental modifications to limit food accessibility) [30]. For example, parents may implement covert restrictions by avoiding the purchase or introduction of unhealthy foods into the household. Meta-analytic evidence [30] indicates that parental overt restrictions are associated with increased food responsiveness and maladaptive eating behaviors in children, whereas covert restrictions show no such adverse effects. Notably, the restrictions scale used in the current study did not distinguish between the two subtypes of restrictions, which may explain the heterogeneous findings. Future research should delineate between restrictions’ subtypes and cultural variations while investigating whether covert restrictions could serve as an alternative strategy for maintaining dietary quality without triggering negative behavioral consequences.

At the within-person level, the RI-CLPM revealed child-driven effects, where increased food fussiness predicted later increases in parental restrictions and pressure to eat. This was consistent with twin studies showing that parents tend to intensify pressure to eat for children exhibiting food fussiness compared with less fussy siblings [31, 32]. However, while these studies found no within-pair differences in restriction related to fussiness, they reported negative between-family correlations between restriction and fussiness [31]. Our RI-CLPM results similarly showed significant negative associations at the between-person level, indicating that households implementing higher restriction exhibited lower overall fussiness. This phenomenon likely occurs because limiting access to palatable energy-dense foods (e.g., snacks, sweets) reduces opportunities for fussiness to manifest. Fussiness primarily involves rejecting nutrient-dense foods such as vegetables and proteins, while parental restriction specifically targets energy-dense nutrient-poor foods including sweets and processed snacks. Conversely, within-person dynamics when persistent fussiness reduces healthy food intake, parents systematically intensify restriction of unhealthy foods as a nutritional compensation strategy to prevent dietary imbalance. These findings collectively suggest that caregivers’ feeding behaviors are reactive adaptations to children’s eating behaviors, probably driven by concerns about weight management and nutritional adequacy [33]. However, from a developmental perspective, such non-responsive feeding behaviors may create negative reinforcement cycles. Although these behaviors may temporarily increase food intake, they could risk diminishing children’s intrinsic eating motivation over time [34].

Compared with the traditional CLPM results, the RI-CLPM in this study did not detect significant effects of parental feeding behaviors on child food fussiness. This may be due to the high genetic stability of food fussiness during the preschool years. Empirical evidence shows that the heritability of food fussiness rises from 60% at 16 months to 74%−84% between ages 3 and 13 [35], reflecting a growing genetic influence on phenotypic variation. Concurrently, the non-heritable component of food fussiness decreases substantially during this window. Therefore, earlier developmental phases, such as infancy or toddlerhood, likely represent a crucial window where environmental inputs could modulate emerging eating patterns. For example, infants exposed to vegetables before 5 months of age had lower fussiness scores [36].

The null findings of parent-driven effects in the RI-CLPM analyses may also stem from methodological constraints. Unlike CLPMs that conflate within-person dynamics with stable between-person differences, the RI-CLPM explicitly isolates within-person fluctuations. This focus, while methodologically rigorous for causal inference, introduces two key constraints affecting interpretation: First, preschool children’s food fussiness exhibits substantial between-individual stability (ICC = 0.52). Consequently, the limited within-person variance remaining after accounting for stable traits in the RI-CLPM reduced statistical power to detect parent-driven effects at the within-person level. This contrasts with CLPMs, which may detect spurious effects driven by between-person correlations rather than true temporal dynamics. Second, parents typically employ multiple types of feeding behaviors simultaneously [37]. Examining isolated dimensions without accounting for their interactive complexities may dilute the observed impacts of feeding behaviors.

Notably, significant cross-lagged effects were observed primarily between T2 and T3, whereas those spanning the T1→T2 interval were markedly less pronounced. This pattern may be explained by developmental trajectories related to fussy eating. In the early stage (T1–T2), picky eating exhibits low temporal stability, for example, in the non-significant autoregressive coefficient from T1 to T2 in the RI-CLPM. During this developmental window, parents often struggle to differentiate transient food refusal from more stable preferences. The convergence of high behavioral variability in young children and limited parental discernment likely reduces the detectability of cross-lagged effects in statistical models during the early childhood period.

The current study did not detect significant moderating effects of child sex in preschool populations. This finding may reflect limited sex-based differences in feeding dynamics during early childhood, as sex differences in food preferences often emerge later in development [14]. Alternatively, caregiver responses to food fussiness, such as restrictive or pressuring feeding behaviors, might operate similarly across sexes in preschool populations due to comparable parental concerns about growth norms at this age [38].

## Implications for practice

The findings of this study, particularly the use of RI-CLPM, underscore the importance of considering both between-person and within-person dynamics in the study of child food fussiness and parental feeding behaviors. Public health initiatives should focus on psychoeducational programs that enhance caregivers’ understanding of typical child eating behaviors and provide proactive strategies for supporting healthy eating patterns. Furthermore, intervention programs should consider the variability of feeding practices across families and the need for tailored approaches that account for individual and family differences.

## Limitations and future directions

Several limitations should be noted. First, the reliance on parental self-report measures for both feeding behaviors and child food fussiness introduces risks of social desirability bias, which may compromise objective assessment. Furthermore, the internal consistency of some feeding behavior subscales, such as the food as a reward subscale (Cronbach’s α < 0.7), may have attenuated the observed associations between these behaviors and child food fussiness. Future research could benefit from using more robust measures or multiple methods to capture dyadic feeding interactions in natural contexts. Second, the sample was predominantly composed of families with higher socioeconomic status (59.3% reporting annual incomes exceeding 500,000 CNY), which limits generalizability to other socioeconomic groups. Comparative studies across income levels and rural-urban divides are necessary to understand how resource accessibility and cultural norms influence feeding dynamics. Third, while sensitivity analyses with complete-case data yielded substantively unchanged results, we acknowledge potential methodological concerns regarding selective attrition. Moreover, given that only a limited number of statistically significant cross-lagged paths were identified, the sample size may have constrained statistical power to detect smaller effects. Future longitudinal studies would benefit from both larger samples and enhanced retention strategies to better address these common methodological challenges in developmental research. Finally, while the study focused on family environments, it did not account for institutional influences such as kindergarten dietary regulations, which may systematically affect children’s eating behaviors.

## Conclusion

The divergent findings between the CLPM and RI-CLPM analyses highlight the critical role of controlling for between-person heterogeneity in longitudinal parent‒child feeding research. After accounting for stable trait-like differences, the RI-CLPM results reframe parental restriction and pressure to eat as reactive responses to child food fussiness rather than caregiver-initiated choices. This paradigm shift has practical significance: community health practitioners and school nutrition specialists should collaborate with caregivers to view feeding challenges through a developmental lens, promoting adaptive parent‒child interactions that foster sustainably healthy eating patterns.

## Supplementary Information

Supplementary Material 1.

## Data Availability

No datasets were generated or analysed during the current study.

## Abbreviations

CLPM: Cross-lagged panel model
RI-CLPM: Random-intercept cross-lagged panel model
BAZ: BMI-for-age Z-scores
MLE: Maximum likelihood estimation
FIML: Full information maximum likelihood
CFI: Comparative fit index
TLI: Tucker‒Lewis index
RMSEA: Root mean square error of approximation
SRMR: Standardized root mean square residual
SD: Standard deviation
ICC: Intraclass correlation coefficients
CI: Confidence interval

## References

[1] Wolstenholme H, Kelly C, Hennessy M, Heary C. Childhood fussy/picky eating behaviours: a systematic review and synthesis of qualitative studies. Int J Behav Nutr Phys Act. 2020;17(1):2. 31900163 10.1186/s12966-019-0899-x PMC6942299

[2] da Costa MP, Severo M, Oliveira A, Lopes C, Hetherington M, Vilela S. Longitudinal bidirectional relationship between children’s appetite and diet quality: a prospective cohort study. Appetite. 2022;169:105801. 34774668 10.1016/j.appet.2021.105801

[3] Dubois L, Bédard B, Goulet D, Prud’homme D, Tremblay RE, Boivin M. Eating behaviors, dietary patterns and weight status in emerging adulthood and longitudinal associations with eating behaviors in early childhood. Int J Behav Nutr Phys Act. 2022;19(1):139. 36384744 10.1186/s12966-022-01376-z PMC9670577

[4] Pereboom J, Thijs C, Eussen S, Mommers M, Gubbels JS. Association of picky eating around age 4 with dietary intake and weight status in early adulthood: a 14-year follow-up based on the KOALA birth cohort study. Appetite. 2023;188:106762. 37385471 10.1016/j.appet.2023.106762

[5] Scaglioni S, De Cosmi V, Ciappolino V, Parazzini F, Brambilla P, Agostoni C. Factors influencing children’s eating behaviours. Nutrients. 2018;10(6):706. 29857549 10.3390/nu10060706 PMC6024598

[6] Berge JM, Miller J, Veblen-Mortenson S, Kunin-Batson A, Sherwood NE, French SA. A bidirectional analysis of feeding practices and eating behaviors in parent/child dyads from low-income and minority households. J Pediatr. 2020;221:93-e9820. 32247517 10.1016/j.jpeds.2020.02.001 PMC7252585

[7] Cole NC, An R, Lee S-Y, Donovan SM. Correlates of picky eating and food neophobia in young children: a systematic review and meta-analysis. Nutr Rev. 2017;75(7):516–32. 28535257 10.1093/nutrit/nux024

[8] Russell CG, Russell A. A biopsychosocial approach to processes and pathways in the development of overweight and obesity in childhood: insights from developmental theory and research. Obes Rev. 2019;20(5):725–49. 30768750 10.1111/obr.12838

[9] Burnett AJ, Jansen E, Appleton J, Rossiter C, Fowler C, Denney-Wilson E, et al. Bidirectional associations between parental feeding practices, infant appetitive traits and infant bmiz: a longitudinal cohort study. Int J Behav Nutr Phys Act. 2022;19(1):153. 36517797 10.1186/s12966-022-01392-z PMC9753278

[10] Jansen E, Williams KE, Mallan KM, Nicholson JM, Daniels LA. Bidirectional associations between mothers’ feeding practices and child eating behaviours. Int J Behav Nutr Phys Act. 2018;15(1):3. 29325557 10.1186/s12966-018-0644-x PMC5765660

[11] Jansen PW, de Barse LM, Jaddoe VWV, Verhulst FC, Franco OH, Tiemeier H. Bi-directional associations between child fussy eating and parents’ pressure to eat: who influences whom? Physiol Behav. 2017;176:101–6. 28215424 10.1016/j.physbeh.2017.02.015 PMC5436628

[12] Mallan KM, Jansen E, Harris H, Llewellyn C, Fildes A, Daniels LA. Feeding a fussy eater: examining longitudinal bidirectional relationships between child fussy eating and maternal feeding practices. J Pediatr Psychol. 2018;43(10):1138–46. 30020501 10.1093/jpepsy/jsy053

[13] Obidoa JC, Onyechi KCN, Chukwuone CA, Dimelu IN, Victor-Aigbodion V, Eseadi C, et al. Gender effect on eating habits of Nigerian school children. Medicine. 2021;100(13):e24961. 33787582 10.1097/MD.0000000000024961 PMC8021307

[14] Qiu C, Hatton R, Li Q, Xv J, Li J, Tian J, et al. Associations of parental feeding practices with children’s eating behaviors and food preferences: a Chinese cross-sectional study. BMC Pediatr. 2023;23(1):84. 36800939 10.1186/s12887-023-03848-y PMC9938626

[15] Hyczko AV, Ruggiero CF, Hohman EE, Anzman-Frasca S, Savage JS, Birch LL, et al. Sex differences in maternal restrictive feeding practices in the intervention nurses start infants growing on healthy trajectories study. Acad Pediatr. 2021;21(6):1070–6. 34020105 10.1016/j.acap.2021.05.002 PMC8349795

[16] Jansen PW, Derks IPM, Mou Y, van Rijen EHM, Gaillard R, Micali N, et al. Associations of parents’ use of food as reward with children’s eating behaviour and BMI in a population-based cohort. Pediatr Obes. 2020;15(11):e12662. 32548949 10.1111/ijpo.12662 PMC7583369

[17] Lucas RE. Why the cross-lagged panel model is almost never the right choice. Adv Methods Pract Psychol Sci. 2023;6(1):25152459231158378.

[18] Hamaker EL, Kuiper RM, Grasman RP. A critique of the cross-lagged panel model. Psychol Methods. 2015;20(1):102–16. 25822208 10.1037/a0038889

[19] Burns RA, Crisp DA, Burns RB. Re-examining the reciprocal effects model of self-concept, self- efficacy, and academic achievement in a comparison of the cross-lagged panel and random- intercept cross-lagged panel frameworks. Br J Educ Psychol. 2020;90(1):77–91. 30657590 10.1111/bjep.12265

[20] Orth U, Clark DA, Donnellan MB, Robins RW. Testing prospective effects in longitudinal research: comparing seven competing cross-lagged models. J Pers Soc Psychol. 2021;120(4):1013–34. 32730068 10.1037/pspp0000358 PMC7854859

[21] Yang X, Jiang X, Zhang Y, Sun L, Wang C, Shang L. Development and evaluation of preschooler’s eating behavior scale. Chin J Child Health Care. 2012;20(8):682–5.

[22] Zheng L, Song D, Chen C, Li F, Zhu D. Reliability and validity of a Chinese version of child feeding questionnaire among parents of preschoolers. Chin J Child Health Care. 2016;24(10):1019–23.

[23] Multicentre Growth Reference Study Group WHO. WHO child growth standards based on length/height, weight and age. Acta Paediatr (Oslo Norway: 1992). 2006;Supplement(450):76–85. 10.1111/j.1651-2227.2006.tb02378.x 16817681

[24] Kline RB. Principles and practice of structural equation modeling. 4th ed. New York, NY, US: Guilford Press; 2016. p. 534.

[25] Farrow C, Blissett J. (2012). Stability and continuity of parentally reported child eating behaviours and feeding practices from 2 to 5 years of age. Appetite. 2012;58(1):151–156. 10.1016/j.appet.2011.09.005 21986188

[26] Powell F, Farrow C, Meyer C, Haycraft E. (2018). The Stability and Continuity of Maternally eported and Observed Child Eating Behaviours and Feeding Practices across Early Childhood. nt J Environ Res Public Health. 2018;15(5):1017. 10.3390/ijerph15051017 PMC5982056 29783638

[27] Shahdan S, Sidek S. The influence of family characteristics on food parenting practices among parents with school-age children and adolescents: a systematic review. Appetite. 2025;210:107979. 40157508 10.1016/j.appet.2025.107979

[28] Gregory JE, Paxton SJ, Brozovic AM. Maternal feeding practices, child eating behaviour and body mass index in preschool-aged children: a prospective analysis. Int J Behav Nutr Phys Act. 2010;7:55. 20579397 10.1186/1479-5868-7-55 PMC2907299

[29] Werner LM, Mallan KM. Associations between restrictive feeding practices and children’s dietary intake: systematic review and meta-analyses. Appetite. 2024;200:107508. 38795944 10.1016/j.appet.2024.107508

[30] Say A, de la Piedad Garcia X, Mallan KM. The correlation between different operationalisations of parental restrictive feeding practices and children’s eating behaviours: systematic review and meta-analyses. Appetite. 2023;180:106320. 36210017 10.1016/j.appet.2022.106320

[31] Harris HA, Fildes A, Mallan KM, Llewellyn CH. Maternal feeding practices and fussy eating in toddlerhood: a discordant twin analysis. Int J Behav Nutr Phys Act. 2016;13:81. 27412445 10.1186/s12966-016-0408-4 PMC4944306

[32] Kininmonth AR, Herle M, Tommerup K, Haycraft E, Farrow C, Croker H, Pickard A et al. (2023). Parental feeding practices as a response to child appetitive traits in toddlerhood and early childhood: A discordant twin analysis of the Gemini cohort. Int J Behav Nutr Phys Act. 2016;20(1):39. 10.1186/s12966-023-01440-2 PMC10074660 37016417

[33] Melbye EL, Hansen H. Child weight and parental feeding practices: a child-responsive model. Nutr Food Sci. 2015;45(1):174–88.

[34] Liew J, Zhou Z, Perez M, Yoon M, Kim M. Parental child-feeding in the context of child temperament and appetitive traits: evidence for a biopsychosocial process model of appetite self-regulation and weight status. Nutrients. 2020;12(11):3353. 33143216 10.3390/nu12113353 PMC7692583

[35] Nas Z, Herle M, Kininmonth AR, Smith AD, Bryant-Waugh R, Fildes A et al. (2025). Nature and nurture in fussy eating from toddlerhood to early adolescence: Findings from the Gemini twin cohort. J Child Psychol Psychiatry. 2025;66(2):241–252. 10.1111/jcpp.14053 PMC11754699 39299707

[36] de Barse LM, Jansen PW, Edelson-Fries LR, Jaddoe VWV, Franco OH, Tiemeier H, et al. Infant feeding and child fussy eating: the generation R study. Appetite. 2017;114:374–81. 28400303 10.1016/j.appet.2017.04.006

[37] Wei X, Wu R, Wang J, Chen J, Tang X, Hua W, et al. A latent class analysis of feeding practices among preschoolers’ parents and its correlations with parental depression status. Chin J Behav Med ༆ Brain Sci. 2023;32(2):152–8.

[38] Loth KA, Mohamed N, Trofholz A, Tate A, Berge JM. Associations between parental perception of- and concern about-child weight and use of specific food-related parenting practices. Appetite. 2021;160:105068. 33352291 10.1016/j.appet.2020.105068 PMC7879703

