# Supplementary material for: Longitudinal associations between food fussiness and parental feeding behaviors in Chinese children: between- and within-person effects
Source: Int J Behav Nutr Phys Act. 2025 Nov 4;22:137. doi: 10.1186/s12966-025-01830-8 (PMC12584304; doi:10.1186/s12966-025-01830-8)
Supplement: Supplementary file 1 — Supplementary Material 1. [file 12966_2025_1830_MOESM1_ESM.docx]

**Table S1. Bivariate correlations among study variables**

|  | 1 | 2 | 3 | 4 | 5 | 6 | 7 | 8 | 9 | 10 | 11 | 12 | 13 | 14 | 15 | 16 | 17 |
| --- | --- | --- | --- | --- | --- | --- | --- | --- | --- | --- | --- | --- | --- | --- | --- | --- | --- |
| **1. Sex** | 1 |  |  |  |  |  |  |  |  |  |  |  |  |  |  |  |  |
| **2. BAZ** | 0.008 | 1 |  |  |  |  |  |  |  |  |  |  |  |  |  |  |  |
| **3. Fedu** | -0.020 | 0.002 | 1 |  |  |  |  |  |  |  |  |  |  |  |  |  |  |
| **4. Medu** | -0.009 | -0.027 | 0.679^***^ | 1 |  |  |  |  |  |  |  |  |  |  |  |  |  |
| **5. income** | 0.003 | 0.008 | 0.193^***^ | 0.173^***^ | 1 |  |  |  |  |  |  |  |  |  |  |  |  |
| **6. Fuss_T1** | -0.070 | 0.018 | -0.012 | 0.006 | -0.009 | 1 |  |  |  |  |  |  |  |  |  |  |  |
| **7. Restr_T1** | -0.001 | 0.031 | 0.036 | 0.037 | 0.037 | -0.056 | 1 |  |  |  |  |  |  |  |  |  |  |
| **8. Press_T1** | -0.051 | -0.15^***^ | -0.042 | -0.069 | -0.001 | 0.176^***^ | 0.085^*^ | 1 |  |  |  |  |  |  |  |  |  |
| **9. Reward_T1** | 0.022 | -0.035 | -0.095^*^ | -0.080 | -0.045 | 0.142^***^ | -0.066 | 0.296^***^ | 1 |  |  |  |  |  |  |  |  |
| **10. Fuss_T2** | 0.026 | -0.020 | -0.012 | 0.004 | -0.002 | 0.546^***^ | -0.164^***^ | 0.092^*^ | 0.095^*^ | 1 |  |  |  |  |  |  |  |
| **11. Restr_T2** | 0.013 | 0.080 | 0.098^*^ | 0.088 | -0.053 | -0.085 | 0.370^***^ | 0.066 | -0.080 | -0.178^***^ | 1 |  |  |  |  |  |  |
| **12. Press_T2** | -0.024 | -0.155^***^ | -0.013 | 0.009 | 0.002 | 0.146^**^ | -0.037 | 0.488^***^ | 0.155^***^ | 0.189^***^ | 0.052 | 1 |  |  |  |  |  |
| **13. Reward_T2** | 0.028 | -0.064 | -0.059 | -0.053 | -0.019 | 0.099^*^ | -0.031 | 0.190^***^ | 0.369^***^ | 0.057 | 0.008 | 0.280^***^ | 1 |  |  |  |  |
| **14. Fuss_T3** | 0.049 | -0.022 | -0.022 | -0.005 | 0.015 | 0.521^***^ | -0.188^***^ | 0.107^*^ | 0.125^**^ | 0.643^***^ | -0.152^**^ | 0.210^***^ | 0.188^***^ | 1 |  |  |  |
| **15. Restr_T3** | 0.004 | 0.057 | 0.087 | 0.094^*^ | 0.023 | -0.063 | 0.325^***^ | 0.005 | -0.080 | -0.055 | 0.484^***^ | 0.043 | 0.047 | -0.143^**^ | 1 |  |  |
| **16. Press_T3** | -0.022 | -0.228^***^ | -0.050 | -0.067 | -0.110^*^ | 0.155^***^ | -0.070 | 0.391^***^ | 0.214^***^ | 0.250^***^ | -0.003 | 0.560^***^ | 0.311^***^ | 0.221^***^ | -0.038 | 1 |  |
| **17. Reward_T3** | -0.008 | -0.139^**^ | -0.044 | -0.019 | -0.071 | 0.106* | -0.142^**^ | 0.213^***^ | 0.297^***^ | 0.179^***^ | -0.116^*^ | 0.202^***^ | 0.354^***^ | 0.186^***^ | -0.014 | 0.420^***^ | 1 |

Note. BAZ: body mass index z-score, Fedu: father’s education, Medu: mother’s education, Income: family annual income; Fuss: fussiness, Restr: restriction, Press: pressure to eat, Reward: food as rewards. T1: Time 1, T2: Time 2, T3: Time 3. ^*^*p* < 0.05, ^**^*p* < 0.01,^***^*p* < 0.001.

**Table S2 Model fit comparison of cross-lagged panel model and random intercept cross-lagged panel model for child food fussiness and parental feeding behaviors**

|  | Parental Feeding Behaviors | Model | AIC | BIC | *χ^2^* | *df* | TLI | CFI | RMSEA | SRMR | *p* | Model Comparison | | |
| --- | --- | --- | --- | --- | --- | --- | --- | --- | --- | --- | --- | --- | --- | --- |
|  |  |  |  |  |  |  |  |  |  |  |  | Δ*χ^2^* | Δ*df* | *p* |
| Child  Food  Fussiness | Restriction | CLPM | 6118.9 | 6220.4 | 61.889 | 4 | 0.651 | 0.907 | 0.154  [0.121, 0.189] | 0.049 | <0.001 |  |  |  |
|  |  | RI-CLPM | 6066.1 | 6180.9 | 3.130 | 1 | 0.949 | 0.997 | 0.059  [0.000, 0.138] | 0.016 | 0.077 | 58.759 | 3 | <0.001 |
|  |  | Adjusted RI-CLPM | 5642.6 | 5798.6 | 17.710 | 21 | 1.012 | 1.000 | 0.000  [0.000, 0.029] | 0.018 | 0.667 | 14.580 | 20 | 0.800 |
|  | Pressure to Eat | CLPM | 6214.5 | 6316.0 | 51.315 | 4 | 0.760 | 0.936 | 0.139  [0.107, 0.174] | 0.041 | <0.001 |  |  |  |
|  |  | RI-CLPM | 6169.6 | 6284.3 | 0.391 | 1 | 1.012 | 1.000 | 0.000  [0.000, 0.091] | 0.005 | 0.532 | 50.925 | 3 | <0.001 |
|  |  | Adjusted RI-CLPM | 5691.1 | 5847.2 | 24.505 | 21 | 0.990 | 0.995 | 0.017  [0.000, 0.041] | 0.020 | 0.269 | 24.114 | 20 | 0.237 |
|  | Food as Rewards | CLPM | 6344.5 | 6446.0 | 58.488 | 4 | 0.683 | 0.915 | 0.140  [0.108, 0.175] | 0.046 | <0.001 |  |  |  |
|  |  | RI-CLPM | 6298.7 | 6413.5 | 0.210 | 1 | 1.021 | 1.000 | 0.000  [0.000, 0.083] | 0.004 | 0.647 | 51.790 | 3 | <0.001 |
|  |  | Adjusted RI-CLPM | 5827.7 | 5983.7 | 13.398 | 21 | 1.031 | 1.000 | 0.000  [0.000, 0.016] | 0.015 | 0.894 | 13.189 | 20 | 0.869 |

Note. AIC: Akaike information criterion, BIC: Bayesian information criterion, *χ^2^*: chi-square, *df*: degrees of freedom*,* TLI: Tucker‒Lewis index, CFI: comparative fit index, RMSEA: root mean square error of approximation, SRMR: standardized root mean square residual. CLPM: Cross-Lagged Panel Model, RI-CLPM: Random Intercept Cross-Lagged Panel Model, Adjusted RI-CLPM: RI-CLPM adjusted for child sex, age, BMI-for-age z-score (BAZ), parental education and family annual income.

**Table S3 Cross-lagged model comparisons of child food fussiness and parental restriction**

| **Paths** | **CLPM** | | **RI-CLPM** | | **Adjusted RI-CLPM** | | **RI-CLPM (n=389)** | |
| --- | --- | --- | --- | --- | --- | --- | --- | --- |
|  | ***β* (SE)** | ***p*** | ***β* (SE)** | ***p*** | ***β* (SE)** | ***p*** | ***β* (SE)** | ***p*** |
| **Cross-lagged paths** |  |  |  |  |  |  |  |  |
| Restriction T1→Fussiness T2 | **-0.104(0.035)** | **0.003** | -0.001(0.063) | 0.984 | -0.006 (0.068) | 0.924 | 0.001(0.068) | 0.989 |
| Fussiness T1 → Restriction T2 | -0.046(0.046) | 0.316 | 0.151(0.112) | 0.177 | 0.123 (0.121) | 0.307 | 0.138 (0.122) | 0.259 |
| Restriction T2 → Fussiness T3 | -0.034(0.034) | 0.325 | 0.031(0.061) | 0.611 | 0.038 (0.063) | 0.548 | 0.037 (0.068) | 0.585 |
| Fussiness T2 → Restriction T3 | 0.044(0.045) | 0.331 | **0.179(0.084)** | **0.033** | **0.183 (0.091)** | **0.044** | **0.182 (0.092)** | **0.049** |
| **Autoregression** |  |  |  |  |  |  |  |  |
| FussinessT1 → FussinessT2 | 0.554(0.039) | <0.001 | 0.145(0.105) | 0.166 | 0.082 (0.113) | 0.465 | 0.121 (0.114) | 0.289 |
| FussinessT2 → FussinessT3 | 0.582(0.035) | <0.001 | 0.239(0.075) | 0.001 | 0.234 (0.079) | 0.003 | 0.218 (0.086) | 0.011 |
| Restriction T1 → Restriction T2 | 0.351(0.041) | <0.001 | 0.054(0.093) | 0.561 | 0.016 (0.098) | 0.867 | 0.022 (0.098) | 0.819 |
| Restriction T2 → Restriction T3 | 0.484(0.043) | <0.001 | 0.256(0.080) | 0.001 | 0.221 (0.085) | 0.009 | 0.214 (0.088) | 0.015 |
| **Residual correlations** |  |  |  |  |  |  |  |  |
| T1 | -0.030 (0.021) | 0.163 | 0.049(0.023) | 0.033 | 0.044 (0.023) | 0.054 | 0.023 (0.026) | 0.374 |
| T2 | -0.051 (0.019) | 0.007 | -0.020(0.027) | 0.443 | -0.025 (0.028) | 0.372 | -0.024 (0.030) | 0.436 |
| T3 | -0.048 (0.016) | 0.002 | -0.015(0.016) | 0.375 | -0.014 (0.017) | 0.404 | -0.010 (0.018) | 0.592 |
| **Random intercept correlation** |  |  | -0.075(0.021) | <0.001 | -0.072 (0.021) | 0.001 | -0.081 (0.024) | 0.001 |

Note. *β*: standardized regression coefficients, SE: standard error**,** Adjusted RI-CLPM: RI-CLPM adjusted for child sex, age, BMI-for-age z-score (BAZ), parental education and family annual income.

**Table S4 Cross-lagged model comparisons of parental pressure to eat and child food fussiness**

| **Paths** | **CLPM** | | **RI-CLPM** | | **Adjusted RI-CLPM** | | **RI-CLPM (n=389)** | |
| --- | --- | --- | --- | --- | --- | --- | --- | --- |
|  | ***β* (SE)** | ***p*** | ***β* (SE)** | ***p*** | ***β* (SE)** | ***p*** | ***β* (SE)** | ***p*** |
| **Cross-lagged paths** |  |  |  |  |  |  |  |  |
| Pressure to Eat T1→Fussiness T2 | -0.003 (0.035) | 0.926 | -0.033 (0.072) | 0.649 | -0.034(0.070) | 0.630 | -0.050 (0.079) | 0.528 |
| Fussiness T1 → Pressure to Eat T2 | 0.070 (0.047) | 0.135 | 0.056 (0.112) | 0.618 | -0.006 (0.124) | 0.960 | 0.072 (0.132) | 0.583 |
| Pressure to Eat T2 → Fussiness T3 | 0.058 (0.031) | 0.065 | 0.067 (0.059) | 0.255 | 0.070 (0.061) | 0.249 | 0.074 (0.063) | 0.241 |
| Fussiness T2 → Pressure to Eat T3 | **0.151 (0.048)** | **0.002** | **0.218 (0.087)** | **0.013** | **0.216 (0.091)** | **0.017** | **0.223 (0.095)** | **0.019** |
| **Autoregression** |  |  |  |  |  |  |  |  |
| FussinessT1 → FussinessT2 | 0.563 (0.040) | <0.001 | 0.149 (0.101) | 0.142 | 0.078 (0.110) | 0.482 | 0.120 (0.113) | 0.290 |
| FussinessT2 → FussinessT3 | 0.572 (0.035) | <0.001 | 0.236 (0.073) | 0.001 | 0.228 (0.076) | 0.003 | 0.215 (0.082) | 0.009 |
| Pressure to Eat T1 → Pressure to Eat T2 | 0.494 (0.041) | <0.001 | 0.166 (0.109) | 0.128 | 0.206 (0.104) | 0.048 | 0.164 (0.123) | 0.009 |
| Pressure to Eat T2 → Pressure to Eat T3 | 0.545 (0.041) | <0.001 | 0.286 (0.083) | 0.001 | 0.295 (0.084) | <0.001 | 0.303 (0.085) | <0.001 |
| **Residual correlations** |  |  |  |  |  |  |  |  |
| T1 |  |  |  |  |  |  | 0.014 (0.028) | 0.616 |
| T2 | 0.093 (0.022) | <0.001 | 0.038 (0.025) | 0.130 | 0.033 (0.025) | 0.192 | 0.042 (0.032) | 0.184 |
| T3 | 0.058 (0.019) | 0.002 | 0.044 (0.027) | 0.108 | 0.038(0.029) | 0.198 | 0.032 (0.019) | 0.095 |
| **Random intercept correlation** | 0.019 (0.016) | 0.252 | 0.029 (0.017) | 0.087 | 0.030 (0.017) | 0.078 | 0.075 (0.029) | 0.009 |

Note*.* *β*: standardized regression coefficients, SE: standard error**,** Adjusted RI-CLPM: RI-CLPM adjusted for child sex, age, BMI-for-age z-score (BAZ), parental education and family annual income.

**Table S5 Cross-lagged model comparisons of parental food as a reward and child food fussiness**

| **Paths** | **CLPM** | | **RI-CLPM** | | **Adjusted RI-CLPM** | | **RI-CLPM (n=389)** | |
| --- | --- | --- | --- | --- | --- | --- | --- | --- |
|  | ***β* (SE)** | ***p*** | ***β* (SE)** | ***p*** | ***β* (SE)** | ***p*** | ***β* (SE)** | ***p*** |
| **Cross-lagged paths** |  |  |  |  |  |  |  |  |
| Food as a Reward T1→Fussiness T2 | 0.026 (0.035) | 0.444 | -0.030 (0.061) | 0.619 | -0.025 (0.062) | 0.680 | -0.065 (0.075) | 0.383 |
| Fussiness T1 → Food as A Reward T2 | 0.062 (0.043) | 0.198 | -0.049 (0.114) | 0.664 | -0.043 (0.124) | 0.729 | -0.017 (0.136) | 0.903 |
| Food as a reward T2 → Fussiness T3 | **0.112 (0.032)** | **<0.001** | 0.063 (0.061) | 0.254 | 0.071 (0.056) | 0.207 | 0.097 (0.060) | 0.105 |
| Fussiness T2 → Food as a Reward T3 | **0.144 (0.051)** | **0.005** | 0.093 (0.097) | 0.340 | 0.096 (0.101) | 0.345 | 0.144 (0.106) | 0.177 |
| **Autoregression** |  |  |  |  |  |  |  |  |
| FussinessT1 → FussinessT2 | 0.560 (0.039) | <0.001 | 0.134 (0.103) | 0.194 | 0.065 (0.112) | 0.564 | 0.085 (0.120) | 0.480 |
| FussinessT2 → FussinessT3 | 0.577 (0.034) | <0.001 | 0.243 (0.074) | 0.001 | 0.237 (0.077) | 0.002 | 0.221 (0.081) | 0.006 |
| Food as a Reward T1 → Food as a Reward T2 | 0.354 (0.043) | <0.001 | 0.068 (0.085) | 0.428 | 0.063 (0.086) | 0.463 | -0.002 (0.102) | 0.987 |
| Food as a Reward T2 → Food as a Reward T3 | 0.341 (0.047) | <0.001 | 0.055 (0.087) | 0.526 | 0.051 (0.088) | 0.564 | 0.018 (0.092) | 0.848 |
| **Residual correlations** |  |  |  |  |  |  |  |  |
| T1 | 0.076 (0.022) | 0.001 | 0.009 (0.023) | 0.689 | 0.015 (0.023) | 0.503 | -0.040 (0.025) | 0.118 |
| T2 | -0.010 (0.020) | 0.613 | -0.036 (0.028) | 0.195 | -0.033 (0.029) | 0.267 | -0.026 (0.032) | 0.427 |
| T3 | 0.024 (0.018) | 0.174 | 0.027 (0.019) | 0.152 | 0.030 (0.019) | 0.114 | 0.051 (0.021) | 0.018 |
| **Random intercept correlation** |  |  | 0.065 (0.021) | 0.002 | 0.063 (0.021) | 0.003 | 0.071 (0.024) | 0.003 |

Note. *β*: standardized regression coefficients, SE: standard error**,** Adjusted RI-CLPM: RI-CLPM adjusted for child sex, age, BMI-for-age z-score (BAZ), parental education and family annual income.

**Table S6 The moderating role of child sex on the relation between parental restriction and child food fussiness [*β* (SE)]**

| **Paths** | | | **CLPM** | | **RI-CLPM** | |
| --- | --- | --- | --- | --- | --- | --- |
|  |  |  | **Boys** | **Girls** | **Boys** | **Girls** |
| **Cross-lagged paths** | | |  |  |  |  |
| Restriction T1→Fussiness T2 | | | -0.066 (0.045) | -0.158 (0.054)**^**^** | 0.038 (0.077) | -0.062 (0.104) |
| Fussiness T1 → Restriction T2 | | | -0.030 (0.062) | -0.069 (0.069) | 0.220 (0.166) | 0.090 (0.153) |
| Restriction T2 → Fussiness T3 | | | -0.045 (0.049) | -0.019 (0.048) | 0.043 (0.094) | 0.029 (0.079) |
| Fussiness T2 → Restriction T3 | | | 0.061 (0.057) | 0.023 (0.072) | **0.287 (0.117)^*^** | 0.095 (0.125) |
| **Autoregression** | | |  |  |  |  |
| FussinessT1 → FussinessT2 | | | 0.619 (0.052)**^***^** | 0.488 (0.058)**^***^** | 0.144 (0.151) | 0.150 (0.149) |
| FussinessT2 → FussinessT3 | | | 0.346 (0.054)**^***^** | 0.360 (0.064)**^***^** | 0.125 (0.113) | 0.332 (0.102)^**^ |
| Restriction T1 → Restriction T2 | | | 0.574 (0.049)**^***^** | 0.591 (0.050)**^***^** | 0.029 (0.111) | 0.088 (0.153) |
| Restriction T2 → Restriction T3 | | | 0.478 (0.057)**^***^** | 0.483 (0.067)**^***^** | 0.180 (0.114) | 0.309 (0.116)^**^ |
| **Residual correlations** | | |  |  |  |  |
| T1 | | | -0.030 (0.036) | 0.030 (0.030) | 0.049 (0.029) | 0.048 (0.029) |
| T2 | | | -0.025 (0.024) | -0.082 (0.029) | 0.013 (0.034) | -0.060 (0.041) |
| T3 | | | -0.072 (0.022) | -0.023 (0.023) | -0.023 (0.023) | <0.001 (0.024) |
| **Random intercept correlation** | | |  |  | -0.075 (0.027) | -0.074 (0.033)^*^ |
| Model comparisons | Constrained | *Δχ^2^/Δdf* | 0.606 | | 0.653 | |
|  | Unconstrained | *p* | 0.774 | | 0.733 | |

Note. ^*^*p* < 0.05, ^**^*p* < 0.01, ^***^*p* < 0.001. Constrained: constrained model with cross-lagged paths or intercepts constrained to be equal across groups; Unconstrained: unconstrained model with cross-lagged paths or intercepts freely estimated across groups.

Unconstrained

**Table S7 The moderating role of child sex on the relation between parental pressure to eat and child food fussiness [*β* (SE)]**

| **Paths** | | | **CLPM** | | **RI-CLPM** | |
| --- | --- | --- | --- | --- | --- | --- |
|  |  |  | **Boys** | **Girls** | **Boys** | **Girls** |
| **Cross-lagged paths** | | |  |  |  |  |
| Pressure to Eat T1→Fussiness T2 | | | 0.003 (0.048) | -0.003 (0.051) | -0.032 (0.088) | -0.028 (0.119) |
| Fussiness T1 → Pressure to Eat T2 | | | 0.073 (0.065) | -0.068 (0.067) | 0.048 (0.177) | 0.062 (0.151) |
| Pressure to Eat T2 → Fussiness T3 | | | 0.018 (0.044) | **0.105 (0.044)^*^** | -0.008 (0.082) | **0.177 (0.089)^*^** |
| Fussiness T2 → Pressure to Eat T3 | | | 0.077 (0.065) | **0.234 (0.070)^**^** | 0.089 (0.126) | **0.333 (0.124)^**^** |
| **Autoregression** | | |  |  |  |  |
| FussinessT1 → FussinessT2 | | | 0.621 (0.052)^***^ | 0.504 (0.060)^***^ | 0.134 (0.145) | 0.164 (0.146) |
| FussinessT2 → FussinessT3 | | | 0.486 (0.059)^***^ | 0.504 (0.057)^***^ | 0.128 (0.113) | 0.315 (0.095)^***^ |
| Pressure to Eat T1 → Pressure to Eat T2 | | | 0.572 (0.050)^***^ | 0.570 (0.049)^***^ | 0.214 (0.148) | 0.125 (0.162) |
| Pressure to Eat T2 → Pressure to Eat T3 | | | 0.515 (0.058)**^*^**^**^ | 0.576 (0.059)^***^ | 0.306 (0.106) | 0.258 (0.131) |
| **Residual correlations** | | |  |  |  |  |
| T1 | | | 0.087 (0.030)^**^ | 0.096 (0.032)^**^ | 0.028 (0.030) | 0.042 (0.042) |
| T2 | | | 0.058 (0.026)^*^ | 0.057 (0.028)^*^ | 0.046 (0.036) | 0.041 (0.043) |
| T3 | | | 0.051 (0.024)^*^ | -0.019 (0.022) | 0.043 (0.025) | 0.010 (0.024) |
| **Random intercept correlation** | | |  |  | 0.059 (0.031) | 0.054 (0.040) |
| Model comparisons | Constrained | *Δχ^2^/Δdf* | 1.001 | | 0.997 | |
|  | Unconstrained | *p* | 0.426 | | 0.436 | |

Note. ^*^*p* < 0.05, ^**^*p* < 0.01, ^***^*p* < 0.001. Constrained: constrained model with cross-lagged paths or intercepts constrained to be equal across groups; Unconstrained: unconstrained model with cross-lagged paths or intercepts freely estimated across groups.

**Table S8 The moderating role of child sex on the relation between parental food as a reward and child food fussiness [*β* (SE)]**

| **Paths** | | | **CLPM** | | | **RI-CLPM** | |
| --- | --- | --- | --- | --- | --- | --- | --- |
|  |  |  | **Boys** | **Girls** | | **Boys** | **Girls** |
| **Cross-lagged paths** | | |  |  | |  |  |
| Food as a Reward T1→Fussiness T2 | | | 0.012 (0.045) | 0.038 (0.053) | | -0.114 (0.082) | 0.058 (0.090) |
| Fussiness T1 → Food as a Reward T2 | | | **0.132 (0.065)^*^** | -0.027 (0.073) | | -0.038 (0.180) | -0.093 (0.158) |
| Food as a Reward T2 → Fussiness T3 | | | **0.123 (0.046)^**^** | **0.098 (0.044)^*^** | | 0.055 (0.082) | 0.076 (0.075) |
| Fussiness T2 → Food as a Reward T3 | | | 0.083 (0.071) | **0.225 (0.073)^**^** | | -0.066 (0.142) | **0.271 (0.134)^*^** |
| **Autoregression** | | |  |  | |  |  |
| FussinessT1 → FussinessT2 | | | 0.622 (0.052)**^***^** | 0.498 (0.060)**^***^** | | 0.104 (0.153) | 0.129 (0.146) |
| FussinessT2 → FussinessT3 | | | 0.317 (0.057)**^***^** | 0.407 (0.065)**^***^** | | 0.121 (0.111) | 0.339 (0.097)^**^ |
| Food as a Reward T1 → Food as a Reward T2 | | | 0.563 (0.048)**^***^** | | 0.592 (0.048)**^***^** | -0.035 (0.122) | 0.227 (0.126) |
| Food as a Reward T2 → Food as a Reward T3 | | | 0.343 (0.069)**^***^** | 0.345 (0.065)**^***^** | | 0.002 (0.120) | 0.157 (0.127) |
| **Residual correlations** | | |  |  | |  |  |
| T1 | | | 0.050 (0.030) | 0.105 (0.032)**^**^** | | -0.033 (0.028) | 0.064 (0.041) |
| T2 | | | -0.008 (0.026) | -0.020 (0.031) | | -0.041 (0.037) | -0.038 (0.042) |
| T3 | | | 0.058 (0.026)**^*^** | -0.014 (0.024) | | 0.043 (0.027) | 0.004 (0.026) |
| **Random intercept correlation** | | |  |  | | 0.081 (0.026)^**^ | 0.040 (0.037) |
| Model  comparisons | Constrained | *Δχ^2^/Δdf* | 1.003 | | | 1.156 | |
|  | Unconstrained | *p* | 0.431 | | | 0.322 | |

Note. ^*^*p* < 0.05, ^**^*p* < 0.01, ^***^*p* < 0.001. Constrained: constrained model with cross-lagged paths or intercepts constrained to be equal across groups; Unconstrained: unconstrained model with cross-lagged paths or intercepts freely estimated across groups.
